# Self-Assembled Monolayers of Copper Sulfide Nanoparticles on Glass as Antibacterial Coatings

**DOI:** 10.3390/nano10020352

**Published:** 2020-02-18

**Authors:** Chiara Gargioni, Mykola Borzenkov, Laura D’Alfonso, Paola Sperandeo, Alessandra Polissi, Lucia Cucca, Giacomo Dacarro, Pietro Grisoli, Piersandro Pallavicini, Agnese D’Agostino, Angelo Taglietti

**Affiliations:** 1 Department of Chemistry, University of Pavia, Viale Taramelli 12, 27100 Pavia, Italy; chiara.gargioni01@universitadipavia.it (C.G.); lucia.cucca@unipv.it (L.C.); giacomo.dacarro@unipv.it (G.D.); psp@unipv.it (P.P.); agnese.dagostino@polimi.it (A.D.); 2 Nanomedicine Center, Department of Physics, University of Milano-Bicocca, Piazza dell’Ateneo Nuovo, 20126 Milan, Italy; mykola.borzenkov@unimib.it (M.B.); laura.dalfonso@mib.infn.it (L.D.); 3 Department of Pharmacological and Biomolecular Sciences, University of Milan, via Balzaretti 9, 20133 Milan, Italy; paola.sperandeo@unimi.it (P.S.); alessandra.polissi@unimi.it (A.P.); 4 Department of Drug Sciences, University of Pavia, Viale Taramelli 14, 27100 Pavia, Italy; pietro.grisoli@unipv.it

**Keywords:** copper sulfide nanoparticles, antibacterial surfaces, photo-thermal effect, hyperthermia, Near InfraRed irradiation

## Abstract

We developed an easy and reproducible synthetic method to graft a monolayer of copper sulfide nanoparticles (CuS NP) on glass and exploited their particular antibacterial features. Samples were fully characterized showing a good stability, a neat photo-thermal effect when irradiated in the Near InfraRed (NIR) region (in the so called “biological window”), and the ability to release controlled quantities of copper in water. The desired antibacterial activity is thus based on two different mechanisms: (i) slow and sustained copper release from CuS NP-glass samples, (ii) local temperature increase caused by a photo-thermal effect under NIR laser irradiation of CuS NP–glass samples. This behavior allows promising in vivo applications to be foreseen, ensuring a “static” antibacterial protection tailored to fight bacterial adhesion in the critical timescale of possible infection and biofilm formation. This can be reinforced, when needed, by a photo-thermal action switchable on demand by an NIR light.

## 1. Introduction

The menace of multi-drug resistant bacterial strains intensifies every day, as a result of the increase in the misuse of antibiotics over the past decades [1,2]. Another weakness of “classical” antibiotics is their limited action against biofilms. Biofilms are strongly resistant sessile microbial communities, which produce an extracellular polymer matrix which embeds the colonies and creates a shield which renders the antibacterial actions of drugs quite ineffective. Biofilm-related infections on nosocomial surfaces are one of the leading causes of medical implant failure, as well of other severe nosocomial diseases. In subcutaneous implants, biofilm forms as a result of bacteria adhesion to surfaces usually in the first 12 h after the implantation, an interval in which prevention of adhesion of planktonic bacterial cells is critical for a successful implantation [3,4].

One way to manage these threats to our health relies on the use of the toolbox of inorganic nanochemistry and material sciences. An enormous number of papers have been published on the surface modification or coating of a variety of materials, with the aim of making them intrinsically antibacterial and with the ability to fight the growth of biofilms. In a huge list of cases, modification and coating were based on an approach involving inorganic chemistry and antibacterial elements such as silver and to a lesser extent, copper [4,5,6,7,8]. Metal ions, in fact, offer multiple ways of interference with bacteria survival and replication (binding and damaging of cell walls, generation of oxygen reactive species, interaction with DNA) and so the generation of mutations able to equip bacterial strains with resistance to these agents is extremely difficult: for example, resistance to silver is rare and develops much slower than that to antibiotics, which usually work with only one mechanism [9].

Inorganic nanoparticles can be used for antibacterial treatment, also exploiting a different feature, namely photo-thermally induced localized hyperthermia [10]. For example, gold nanorods (GNRs) [11] and gold nanostars (GNSs) [12] have been proposed to exploit this property: gold itself does not possess antibacterial features, but gold nano-objects featuring intense Localized Surface Plasmonic Resonance (LSPR) bands can achieve thermal relaxation upon proper irradiation, resulting in a localized hyperthermia capable of ablating bacteria in their surroundings. When the absorption band of inorganic nanoparticles is into the so called “biological window” (750–950 nm) in which biological fluids and tissues are semi-transparent, irradiation with a laser could be used, in principle, to treat subcutaneous surfaces. In the last years we realized several models of active surfaces based on this strategy, using self-assembled monolayers (SAM) of gold nanostars or silver nanoplates grafted onto bulk glass samples. The LSPR features of these nano-objects were tailored, by means of shape and dimension selection, to yield LSPR bands placed into the biological window. As a result, NIR laser induced pronounced hyperthermia was efficient to kill bacteria cells both in planktonic states and organized in biofilms [1,13,14].

Amongst inorganic nano-objects offering photo-thermal effects caused by NIR absorption, copper sulfides of general formula Cu_2−x_S (0 < x < 1) stand indeed as a recent and peculiar success, with features which have been extensively described in the last decade [15]. Cu_2−x_S materials have attracted a real interest because of their semiconducting properties and their possible catalytic, photovoltaic and chemosensing applications, to name a few [15,16]. In addition to these features, Cu_2−x_S NP, which are p-type semiconductors, are able to absorb light in the NIR by means of an LSPR phenomenon which is given by collective oscillation of their holes, instead of electrons which act as carriers in noble metal NP. This means a substantial difference when comparing Cu_2−x_S NP to noble metal NP giving LSPR phenomena: in the former case the LSPR position can be regulated by modulation of the number of carriers, which can be obtained changing the x value, i.e., by regulation of Cu_2−x_S stoichiometry. These features have pushed the use of CuS (x = 1) based nanostructures as photothermal agents for cancer cells and bacteria ablation, as CuS covelite nanostructures usually absorb with a maximum in the 950–1000 nm region [16,17,18,19,20,21]. Recently, a study on photothermal and photodynamic effects of ultrasmall laser-activable CuS NP able to eradicate bacteria and to release Cu^2+^ to accelerate wound-healing process was reported [22]. However, a very limited number of studies on the recognized antibacterial effects of CuS NP in the absence of laser irradiation and devoted to exploit effects of CuS NP and Cu^2+^ ions to damage bacterial cells has been reported [23,24,25]. Moreover, no examples are available, to our knowledge, reporting on the use of CuS NP for functionalization of bulk surfaces in order to impart antibacterial abilities to large areas, if we exclude a recent example of polyacrylonitrile fibers coated with CuS [26]. This is quite surprising, as this nanomaterial is considered to have low toxicity [27] and is almost inexpensive, so its use for antibacterial coatings can be recommended. For all these reasons we decided to investigate the antibacterial features of CuS NP SAM grafted onto bulk glass surfaces, in order to exploit, with the same material, two complementary antibacterial actions: (i) the “static” one offered by the microbicidal action of CuS NP and of Cu^2+^ in absence of any laser irradiation, expected to kill planktonic bacteria in the close surroundings of the surfaces of medical devices in the first hours of contamination, preventing their adhesion; (ii) the “on demand” laser-switchable ablation of bacterial cells caused by the photo-thermal effects offered by CuS NP plasmonic features, which could be used, for example, in all cases in which extremely aggressive infections on surfaces cannot sufficiently be controlled by the first mechanism. This is the first time in which the two possible antibacterial actions of this nanomaterial have been studied together exploring the possibility to use them in synergy. The idea is to obtain a prototype of a new, safe and cost effective nanocoating designed to fight bacterial adhesion and proliferation on surfaces, a type of coating that could be strategic to avoid infections, for example, during the first crucial 12–24 h after an implant of a medical device.

## 2. Materials and Methods

### 2.1. Materials

Sodium sulfide anhydrous, sodium citrate dihydrate (≥99%), hydrochloric acid (≥37%), nitric acid (≥65%), sulfuric acid (95%), hydrogen peroxide (30 wt%), ethanol (≥99.7%), (3-aminopropyl)trimethoxysilane (APTES) (≥98.0%), sodium chloride (99%), PBS buffer, were purchased from Aldrich. Copper chloride dihydrate was purchased by FINE CHEMICALS-Atsfaar S.p.A. Microscopy cover glass slides 21 × 26 mm were purchased from DEL Chimica. All reagents were used as received. All the preparation were made with bi-distilled water.

Tryptone Soya Broth (TSB) and Tryptone Soya Agar (TSA) for bacteria culture were purchased from Oxoid, England. *Staphylococcus aureus* ATCC 6538 and *Escherichia coli* ATCC 10536 bacterial strains were used.

### 2.2. Methods

CuS NP synthesis. CuS NPs were synthesized using a slight modification of a reported method [19]. Briefly, to 100 mL of a 1.0 × 10^−3^ mol/L solution of CuCl_2_x 2H_2_O contained in a 250 mL flask, the proper quantity of citrate was added, in order to obtain the desired final concentration (3.4 × 10^−3^, 7.0 × 10^−4^, 3.4 × 10^−4^ mol/L). Under vigorous magnetic stirring, 100 μL of a 1.0 mol/L solution of Na_2_S was added. The solution immediately turned to a dark yellow-brown color. After 5 min of stirring at room temperature, the flask was transferred in a water bath pre-heated at 90 °C and kept for 15 min under stirring. After this time the colloidal suspension turned green, the flask was cooled in an ice bath and after this stored in a refrigerator at 4 °C for maximum 15 days.

Glassware Pre-treatment. All the glassware was usually pre-treated before use: a wash in aqua regia for 30 min, then washed and filled with bi-distilled water and sonicated for 3 min before discarding the water. The bi-distilled water/ultrasound treatment was repeated three times. Then the glassware was dried in an oven for 1 h at 140 °C. Before APTES grafting, microscopy cover glass slides (21 × 26 mm) were treated with piranha solution (3:1 sulfuric acid 95% and hydrogen peroxide 30 wt%) for 30 min. Then the slides were washed in bi-distilled water under sonication for three minutes, three times. After this the glasses were placed in an oven at 140 °C for 1 h.

Functionalization of slides with APTES. The pre-treated cover glass samples were fully immersed in a solution of APTES 10% (v/v) in ethanol and allowed to react for 5 min at 60 °C, using a Hellendhal type glass staining jar. Typically, 8 slides were coated in a single jar. The amino-modified glasses were washed three times with ethanol under sonication. After these steps, the samples were gently dried under N_2_ flux.

Preparation of CuS NP-glass samples. APTES coated glass slides were fully immersed in a CuS NP colloidal suspension, using a Hellendhal type glass staining jar, and kept for 12 h at a controlled temperature of 25 °C under gentle shaking with a Heidolph Promax 1020 reciprocating platform. Typically, 8 slides were coated in a single jar. After immersion, the slides were washed three times in water and carefully dried under a N_2_ stream. Dried samples were stored in the dark in a desiccator and were found to be stable for at least three months.

### 2.3. Characterizations

UV-Vis Spectroscopy. UV-Vis-NIR absorption on colloidal samples and functionalized slides was routinely measured using a Varian Cary 100 UV/Vis spectrophotometer. The wavelength scan range was 300–1100 nm. The liquid samples were measured in quartz cuvettes with 10 mm optical path. The solid samples were placed in a special holder enabling transmission measurement of the same spot on the slide during all experimental stages. For larger range spectra (300–1800 nm) a Varian Cary 6000 I was used.

Transmission electron microscopy (TEM). TEM images of a representative sample of CuS NP were taken on a Jeol JEM-1200 EX II instrument on 1:10 diluted CuS NP solution, with a 10 μL sample dropped on Copper grids (300 mesh) coated with a Parlodion membrane.

Dynamic light scattering (DLS) Diameter and Z-potential of CuS NP colloidal suspensions were routinely measured with a Zetasizer Nano ZS90 Malvern instrument, equipped with dedicated cuvettes. Measurements were repeated at least three times for each sample using 1 mL of colloidal suspension for each one.

Contact Angle. Static contact angle determinations were made with a KSV CAM200 instrument, with the water sessile drop method.

Scanning Electron Microscopy (SEM). SEM images were taken from Tescan Mira XMU variable pressure Field Emission Scanning Electron Microscope—FEG SEM (Tescan USA Inc., Warrendale, PA, USA) located at the Arvedi Laboratory, CISRiC, Pavia. Slides were mounted onto aluminum stubs using double sided carbon adhesive tape and were then made electrically conductive by coating in a vacuum with a thin layer of Pt/Pd (3–5 nm). Observations were made in backscattered electrons mode (BSE) at 30 kV and with InBeam secondary electron detector for higher spatial resolution.

Raman Spectroscopy. Measurements were carried out at room temperature by using a Labram Dilor spectrometer equipped with an Olympus microscope HS BX40. The 632.8 nm light from a He–Ne laser was employed as excitation radiation. The samples, mounted on a motorized xy stage, were tested with a 50x objective and with a laser spot of 2 μm of diameter. Power was set to 10 mW. The spectral resolution was about 1 cm^−1^. Typical values for the power density were about 5 × 10^5^ W/cm^2^.

Determination of copper concentration on surfaces and of copper release in solution. The total Cu content brought on Cu NP-glass was determined by quantitatively oxidizing the CuS NP grafted on a single slide (21 × 26 mm coated on both sides, total coated surface 10.92 cm^2^) by dipping it in 3 mL ultrapure concentrated HNO_3_ diluted 1:5 with water in a beaker, and keeping it overnight at RT on a HeidolphPromax 1020 reciprocating platform shaker. The Cu content in solution was then determined by inductively coupled plasma optical emission spectroscopy (ICP-OES) with an ICP-OES OPTIMA 3300 DV Perkin Elmer instrument. The measure was repeated on 10 glass samples coming from 5 different preparations (two for each preparation).

Release of copper versus immersion time was followed on a set of 5 Cu NP-glass slides (21 × 26 mm coated on both sides, total coated surface 10.92 cm^2^) prepared as described above. Each slide was then immersed in 3 mL of ultrapure water. Slides were taken off the water after 1 h, 5 h, 24 h, 48 h, and 168 h. A UV-Vis spectrum was measured on the obtained solution to check for presence of CuS NP spectral features, and then, after acidification with a few drops of ultrapure concentrated HNO_3_, the content of released copper was determined by ICP-OES. The experiment was repeated three times, and mean values were calculated. ICP data were collected with an ICP-OES OPTIMA 3300 DV Perkin Elmer instrument.

Photo-thermal effect of CuS monolayers upon NIR light irradiation. The monolayers were irradiated with NIR light at a wavelength range 800–1000 nm (Mai Tai, Spectra Physics, Mountain View, CA, USA). The diameter of the beam spot was set as ~13 mm. The substrate temperature changes were monitored by means of a thermo-vision camera (FLIR, E40, USA) and the supporting analysis software.

Measure of Microbicidal Effect (ME) The antibacterial activity of functionalized cover glasses was investigated against S. aureus ATCC 6538 (Gram+) and *E. coli* ATCC 10356 (Gram-). The microorganisms were grown overnight in Tryptone Soya Broth (Oxoid; Basingstoke, Hampshire, England) at 37 °C. Washed cells were re-suspended in Dulbecco’s PBS and optical density (OD) was adjusted to 0.2 at 650 nm wavelength, corresponding approximately to 1 × 10^8^ colony forming units (CFU)/mL. Then, 10 μL of bacterial suspension was deposited on a standard glass slide (76 × 26 mm), and the microbial suspension was covered with a CuS NP-glass sample (21 × 26 mm), forming a thin film between the slides that facilitated direct contact of the microorganisms with the active surface. The two assembled glasses were introduced in a Falcon test-tube (50 mL) containing 1 mL of PBS to maintain a damp environment. In this test, microbes are incubated in non-nutritive suspensions that do not give the microorganisms the potential to grow during the test. For each bacterial strain two equivalent modified glasses were prepared; the slides were maintained in contact with the liquid films containing bacteria at room temperature for 5 and 24 h; for each time of contact an unmodified blank glass slide was treated in the same way as the control sample. After the times of contact, 9 mL of PBS was introduced in each Falcon test-tube under gentle shaking to detach the assembled glass slides. Bacterial suspensions were then cultured on Tryptone Soya Agar (Oxoid; Basingstoke, Hampshire, England) to count the viable cells. The decimal-log reduction rate, i.e., the Microbicidal Effect (ME), was calculated using the formula:

ME = log N_C_ − log N_E_

(N_C_ being the number of CFU/mL developed on the unmodified control glasses, and N_E_ being the number of CFU/mL counted after exposure to modified glasses). The results expressed as ME represent the average of three equivalent determinations. This method can be considered as a version of the Japanese Industrial Standard JIS Z 2811 developed to measure the antibacterial activity of plastic surfaces.

Antibacterial effect triggered by NIR light irradiation. Pseudomonas *aeruginosa* PAO1 strain was selected as model of opportunistic pathogen Gram-negative bacteria. Bacteria were routinely grown in Muller-Hinton (MH, Difco; Franklin Lakes, NJ, USA) agar plates. For each experiment, a single bacterial colony was inoculated in 3 mL of liquid MH broth and incubated at 37 °C under shaking for 15 h. Bacterial concentration was estimated by spectrometric measurement (V-530, Jasco, Tokyo, Japan) at 600 nm, considering that 2 × 10^8^ CFU/mL approximately correspond to an OD600 = 1. Concentration was adjusted to 5 × 10^7^ CFU/mL by diluting bacterial suspensions in fresh MH broth. Monolayers on glass were cut to fit laser beam size and placed in Petri dishes with a cover glass bottom (MatTek, Ashland, MA, USA). An amount of 20 μL of diluted bacterial suspension was inoculated on the top of the glasses, and the films were gently air-dried under laminar flow hood. The samples were irradiated with 950 nm laser with different irradiation durations (30 and 60 min). The non- irradiated samples, the blank, and CuS NP functionalized, were used as control. Irradiated and non-irradiated (control) samples were stained with Film Tracer Live/Dead viability kit (L10316, Invitrogen, Carlsbad, CA, USA, NL) based on the use of the SYTO^®^ 9 and propidium iodide stains mixture in an appropriate concentration ratio (0.167 or 3.34:20) to ensure that the bacteria with intact membranes (live) stain fluorescent green (SYTO^®^ 9) whereas propidium iodide stains red only the bacteria with damaged membranes (dead) [28]. The stained samples were analyzed with a Leica SP5 TCS confocal microscope using a 20x dry objective (HC PL FLUOTAR 20x 0.5, dry, Leica, Germany). At least three z-stack of images were collected from three distant regions in the film by using the 488 nm argon ion laser emission in both spectral intervals (510–580 nm and 590–700 nm, where the bleed through of the green dye at the concentration used for the staining experiments is negligible). The images were analyzed by employing a threshold filter (Fiji, NIH, which was developed at National Institute of Health USA) and measuring the percentage area in the red (A_dead_) and green (A_live_) channels.

## 3. Results and Discussion

### 3.1. CuS NP Synthesis

CuS NP were synthesized in aqueous solution using a slight modification of a reported method, by reacting CuCl_2_ and Na_2_S in the presence of sodium citrate at 90 °C for 15 min [19]. As was clearly demonstrated [19,20] this synthetic pathway produces pure covelite CuS phase, a fact that we checked by means of Raman spectroscopy observing the expected peak at 472 cm^−1^ (see supplementary information, Figure S1) [29]. The synthesis was repeated setting sodium citrate concentration to three different values, in order to modulate the NP dimensions. Investigation with Dynamic Light Scattering and UV-Vis-NIR spectrophotometer gave the results which are summarized in Table 1.

**Figure 1:**
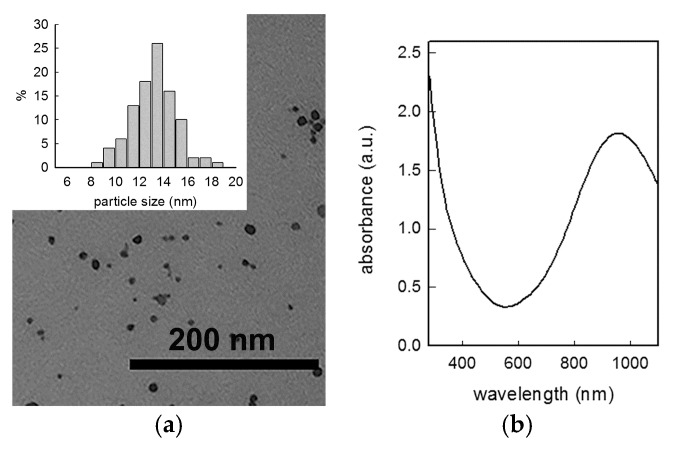
(**a**) Representative TEM image with histogram of size distribution (inset) and (**b**) UV-Vis-NIR spectrum of CuS NP obtained with 7 × 10^−4^ M citrate concentration.

As could be expected, the quantity of citrate, which acts as a capping agent, regulates the dimension of NP obtained, with an increase of dimensions (and decrease of overall colloidal surface area) when the concentration of the capping agent decreases. Z-potential was found, as expected, at a moderately negative value (<−25 mV). UV-Vis-NIR spectra confirmed the presence of an intense LSPR absorption placed in the NIR, with a maxima placed in the expected 900–1000 nm range (see Figure 1). It is interesting to note that the decrease of the NP size is reflected in a small red shift of the LSPR absorption, as previously noticed by Luther et al. [30]. Colloidal suspensions are almost stable in the reaction mixture conserved at 4 °C, but only for few days, as the LSPR band consistently decreases in intensity with time.

TEM images (see Figure 1) were registered for the preparation yielding the CuS NP showing the intermediate dimension as measured from DLS studies: analysis of images gave a mean diameter of 13 nm, confirming the value obtained by DLS.

### 3.2. Glass Functionalization

**Scheme 1:**
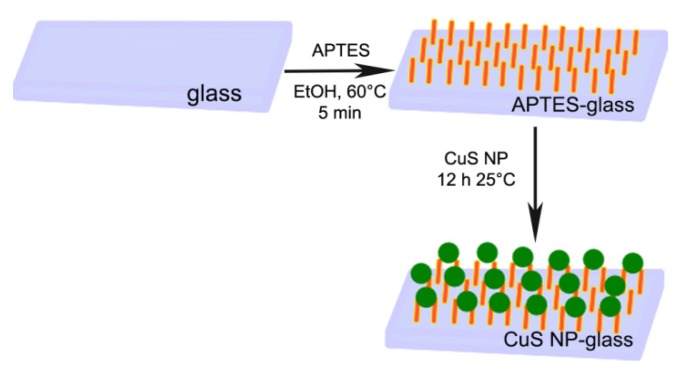
Schematic representation for the preparation of CuS NP-glass samples.

Microscope slides were functionalized with APTES, using a well-established methodology [7]. To obtain the deposition of a monolayer of CuS NP, APTES-terminated glass samples were simply immersed in the native colloidal suspension for increasing time intervals, and then a spectrum of the glass sample was taken. Immersion longer than 12 h did not produce any increase in glass absorbance and so this interval time was set as standard immersion time. A representation of the preparation procedure is reported in Scheme 1.

**Figure 2:**
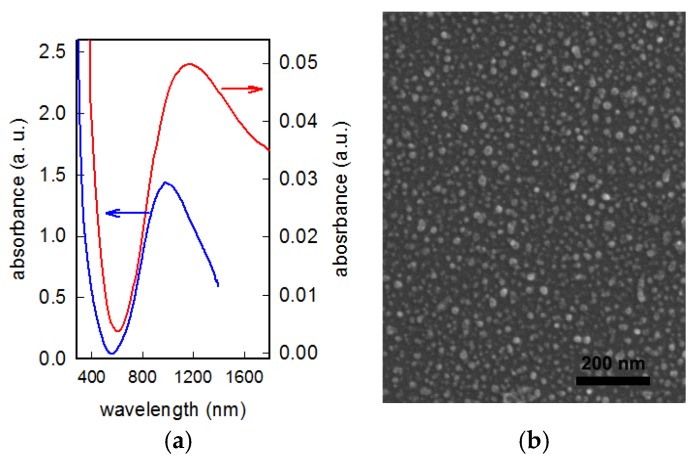
(**a**) UV–Vis spectrum of a CuS NP-glass sample (red line, absorbance on right axe) compared with the spectrum of the colloidal suspension used for functionalization (blue line, absorbance on left axe); (**b**) SEM image of the CuS NP-glass sample.

After immersion, samples were carefully washed three times in order to remove the presence of multilayers or aggregates bound to the surface in a labile fashion, and thus to ensure the presence of a homogeneous monolayer. Reproducibility of the functionalization was assessed by UV-Vis-NIR spectra, by contact angle measurements and by determination of the quantity of Cu found by ICP (vide infra). Formation of the CuS NP monolayer with grafting on APTES functionalized glass can be ascribed to electrostatic interactions: the pH of CuS NP colloidal suspensions is around the value of 6.5, and at this pH the amino functions brought on glass by APTES functionalization are mostly protonated and able to bind the citrate capped CuS NP which offer their negative charge. Contact angles of samples moved from the value of 55 (5)° observed for APTES terminated slides to the value of 90 (5)° after CuS NP grafting. No literature data is so far available on CuS NP layers, so the contact angle values cannot be compared, nevertheless they quickly and clearly account for surface functionalization, as confirmed by electronic microscopy. SEM images of the functionalized glass samples show a homogeneous, uniform layer of CuS NP (see Figure 2). Raman spectra on a CuS NP-glass slide sample confirmed the presence of CuS in the covellite phase (see supplementary information, Figure S1).

The most surprising finding, however, was the fact that the LSPR peak was consistently red shifted, with a maximum at a wavelength out of the range of our routinely used spectrophotometer. The spectrum of a 12 h immersed glass sample, compared with the spectrum of the colloidal suspension used for the functionalization, is reported in Figure 2a. As can be clearly observed, the LSPR band maximum for the monolayer on CuS NP-glass samples undergoes a consistent red-shift of about 200 nm, moving to 1170–1190 nm. SEM images demonstrate that the morphology and size of CuS NP did not change upon grafting on glass, as can be seen in Figure 2b (for a SEM image of an APTES functionalized glass in absence of CuS NP, see Figure S2). Calculation of mean diameter in this case gave a value of 14 (3) nm, in good agreement with DLS and TEM evidences.

The red shift observed in LSPR features when moving from the colloidal suspension to the grafted monolayer can be tentatively explained only with the presence of plasmonic coupling [31,32] resulting from the dense arrangement of CuS NP on the surface. To our knowledge, there are no examples of studies on CuS NP arrays grafted on this kind of surface, and some further investigation is needed to fully understand this LSPR shift phenomena.

From SEM images, the surface density of NP on the monolayer can be calculated to be close to 4 × 10^11^ NP/cm^2^. Considering a monolayer of NP with mean diameter of 13 nm and using the density of bulk CuS (4.76 g/cm^2^), the surface concentration of Cu can be estimated to be close to the value of about 1 μg/cm^2^. This roughly calculated value is close to the experimentally determined one, which was obtained by measuring with ICP the total amount of copper in solution after treatment of glass samples with concentrated nitric acid: the mean value (obtained on repetition of measure on 10 glass samples, confirming a good reproducibility of the preparation) is 0.5(1) μg/cm^2^. Consistency of the value calculated from counting NP surface density in SEM images with the one coming from ICP detection clearly rule out the presence of multilayers.

### 3.3. Copper Release in Water

**Figure 3:**
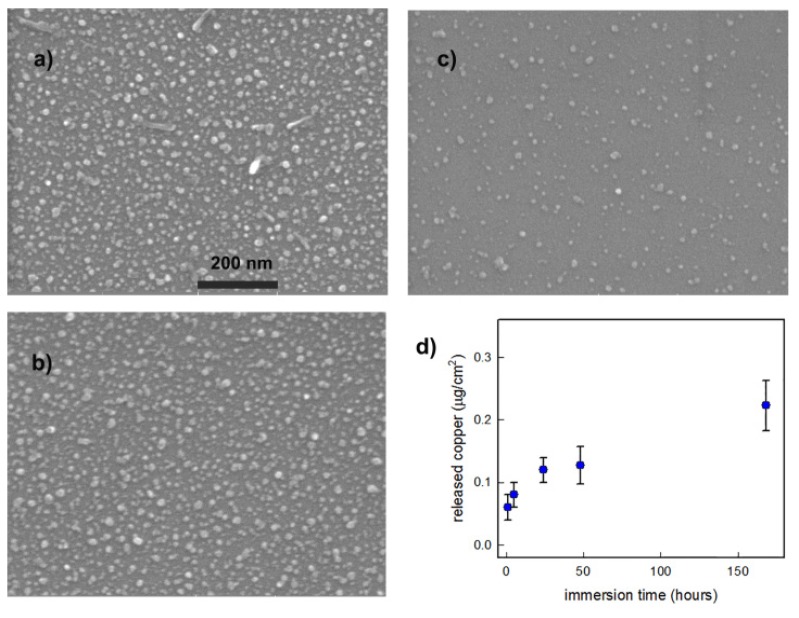
Behavior of CuS NP-glass samples upon immersion in water: (**a**) SEM image of a portion of a standard sample soon after preparation; (**b**) SEM image of a portion of the same sample after 24 h of immersion in water; (**c**) SEM image of a portion of the same sample after one week of immersion in water; (**d**) copper released from standard 21 × 26 mm samples in 5 mL of distilled water at different times (max: 1 week) expressed as micrograms released per cm^2^ of surface(samples mean copper surface concentration: 0.5 (1) μg/cm^2^).

Immersion in a controlled amount of ultrapure water (3 mL) of a standard CuS NP-glass sample was performed in order to assess the quantity of copper released as a function of immersion time. The glass slides were immersed for 1, 5, 24, 48, and 168 h, and after this time removed from the liquid. The UV-Vis-NIR spectra of solutions obtained after immersion were taken, demonstrating the absence of spectral features typical of CuS NP, allowing to rule out the release of NP. Solutions were subsequently analyzed with ICP to detect the quantity of copper released in solution. The experiment was repeated three times. The results are reported in Figure 3d, where the copper released from slides is expressed as micrograms released per cm^2^ of surface, to be compared with the mean total quantity of copper loaded previously indicated, which was 0.5(1) μg/cm^2^. After 24 h we found that about 20% of copper loaded on the surface was released, a value reaching about 50% after one week of immersion. To complete this measure, one freshly prepared sample was divided into three pieces, and three SEM images were taken (Figure 3a–c): one soon after preparation, one after 24 h of immersion, and one after one week of immersion in water. No evident changes could be observed for the 24 h immersed sample, apart from a hardly detectable degree of consumption of NP. After one week the dimensions seem sensibly reduced, and also the number of grafted NP seemed to be decreased: at this point it is hard to assess if this has to be ascribed to consumption of smaller objects or also to detachment of some nanoparticles, which anyway were not detected with the spectrophotometer on the resulting solution. We must stress that these experiments were performed in ultrapure water, in analogy with previous experiments performed on Ag nano-objects [7,13], thus these measurements can be judged as only qualitative, especially when planning to move to biological media where biomolecules could give chelating complexes with copper ions. Nevertheless, these experiments indicate that the investigated samples were capable of a slow release of very small quantities of copper ions upon immersion in water (well below 0.5 mg/L in the conditions of the release). These findings suggest this sustained release could be used for antibacterial purposes while at the same time not being harmful to human cells: as recently demonstrated, CuS nanoparticles releasing similar quantities of Cu(II) ions can promote wound healing while producing antibacterial activities [22]. Copper is released as a function of immersion time, giving a constantly increasing concentration for at least a week, with a sensible quantity released in the first 24–48 h of immersion (without NP detachment), a timescale which usually is critical for fighting bacterial proliferation. After immersion in water for one week we also noticed a strong decrease in the intensity of plasmonic absorption and a blue shift of the weakened plasmonic band, which tends to go back below 1000 nm (see Figure S3). This blue shift can be explained by reduced plasmonic coupling, as the more the particles decrease in dimensions (in some cases reaching complete dissolution, see Figure 3c), the less they can interact to give plasmonic coupling.

### 3.4. Photo-Thermal Effect of the Samples under NIR Light Irradiation

**Figure 4:**
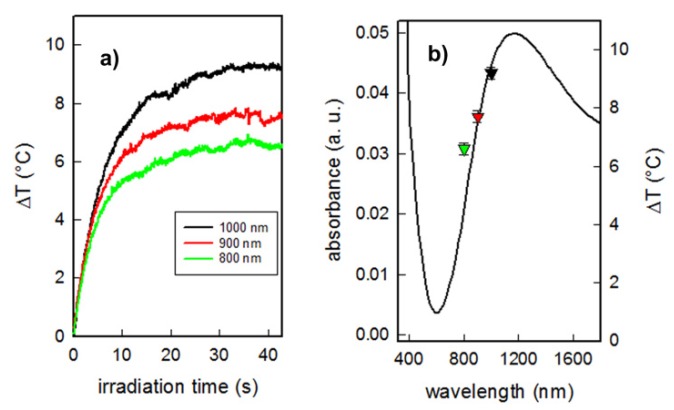
(**a**) Temperature increase as a function of irradiation time, measured under NIR laser irradiation at different wavelengths (800–1000 nm, 0.26 W/cm^2^) of Cu NP-glass samples; (**b**) photo-thermal effect as increase of temperature (right axe) of Cu NP-glass samples, as a function of irradiation wavelength (each color of point corresponds to a wavelength as in panel (a)), superimposed to spectrum of the functionalized glass sample.

After the characterization of the monolayers, we evaluated the ability of CuS NP-glass samples to produce photo-thermal effects when properly irradiated with an NIR laser source. The samples were irradiated at 800, 900, and 1000 nm with laser power 300 mW corresponding to laser intensity 0.26 W/cm^2^, a value which is considered safe for exposure of the skin, well under the proposed limit of 0.32 W/cm^2^ [33]. Unfortunately, due to the limitation of laser source operation wavelengths we could not apply the wavelength corresponding to maximum absorbance of CuS monolayers. Therefore, we focused at first on the NIR window. In all cases a rapid temperature increase reaching a plateau within 15–20 s was observed (see Figure 4a).

Such temperature increase is typical as previously observed for monolayers of gold [14] and Prussian Blue [34] nanoparticles. As already noticed, no temperature increase was observed while irradiating bare “blank” glasses (without CuS NP). Increasing the irradiation wavelength and approaching to a maximum of the LSPR band leads to an increase of localized photo-thermal effect (see Figure 4b). The maximum temperature increase (ΔT 9 °C) was observed under irradiation at 1000 nm. We have already demonstrated and commented on how this weak but localized hyperthermia can be exploited to kill bacteria [1]. We did not measure the photo-thermal effects upon irradiation of samples after prolonged immersion in water, as we noticed a strong decrease in the plasmonic NIR absorption band after one week immersion in water (see Figure S3). We can expect that a very small hyperthermia is obtained upon laser irradiation of these samples, as temperature increase is a function of absorbance. However, we did not consider this fact to be a problem, as these coatings are intended to exert their antibacterial action (based on copper release and/or on laser induced hyperthermia) in the first, critical hours of the use of medical device surfaces.

### 3.5. Antibacterial Behavior in Absence of Irradiation

The microbicidal effect (ME) of CuS NP-glass samples against *E. coli* and *S. aureus*, taken as representative strains of Gram- and Gram+ bacteria respectively, was investigated. In the last decade we introduced a procedure to measure the ME of a functionalized surface when placed in contact with a thin film of bacterial suspension. This method gives a measure of how much, in a given interval, the contact with a microbicidal surface reduces the bacterial survival and proliferation. The ME can be calculated using the formula:

ME = log NC − log NE(1)

where NC is the number of CFU/mL developed by a bacterial suspension after contact with a blank, not functionalized control glass sample, and NE the number of CFU/mL counted after exposure to modified glass samples (CFU = colony forming unit).

We choose the two contact times of 5 and 24 h and evaluated the ME after these intervals, which are reported in Table 2. In ME measurements the longer contact time is usually set to 24 h, as biofilms colonies tend to grow on surfaces in the first 12–18 h following the bacterial contamination; an antibacterial activity working for at least 24 h on planktonic bacteria is a requirement to impart to biomedical surfaces the ability to prevent the initial phase of biofilm growth [7]. Results are reported in Table 2.

Data obtained show that CuS NP-glass have a good ME which increases with contact time, as already observed for surfaces coated with AgNP, Ag(I) or Cu(II) containing moieties. Elimination of bacteria is obtained with a value higher than 99.9% after a contact time of 24 h for both strains, but more than 95% of cells are eliminated after the first 5 h, confirming the ability of surfaces to efficiently kill both representative strains in this timescale [4,5,7,14]. The antibacterial action is reasonably due to copper release, and was largely expected [35]: nevertheless, to our knowledge, this is the first report in which a bulk surface has been rendered antibacterial simply by grafting of CuS NP, a safe, cost effective and easy to prepare nanomaterial.

### 3.6. Photo-Thermal Antibacterial Action

CuS NP are indeed much more renowned for their photo-thermal abilities, and these features have been widely used also for antibacterial purposes: as cited above no examples are present, to our knowledge, on the use of CuS NP to functionalize bulk surfaces exploiting both the static, copper-related action, and the on demand action given by photothermal effects.

Thus, we moved to evaluate the antibacterial action resulting from the localized photo-thermal effect upon NIR light activation. In these experiments, *P. aeruginosa* cells were inoculated onto the glasses and exposed to NIR laser irradiation. *P. aeruginosa* is known to be one of the most common opportunistic pathogens and with high adaptability to environmental changes [36]. Therefore, it is essential to develop new and light-weight approaches aimed at efficient eradication of such type of bacteria. We set the value of irradiation wavelength to 950 nm, in the upper limit of the first biological window. In order to keep the obtained maximum temperature increase close to 10 °C we raised the power to 450 mW, reaching an irradiance of 0.35 mW/cm^2^, a value which is still close to maximum allowing for exposure of the skin (0.32 W/cm^2^) [33]. In these conditions we obtained the thermogram reported in Figure S4. We did not irradiate blank glass with the laser as in previous papers [1,13] we demonstrated that even high laser intensities cannot kill bacteria in the absence of photo-thermal effects. The samples were irradiated continuously for 30 and 60 min. They were then stained and analyzed by means of confocal microscopy and compared to the control samples (non-irradiated inoculated films, both blank and functionalized). We segmented the red emitting (propidium iodide stained, dead) cells and the green emitting (SYTO^®^ 9 stained, live) cells, and the antibacterial effect was measured as the ratio of red to the green area of the image A_dead_/A_live_.

**Figure 5:**
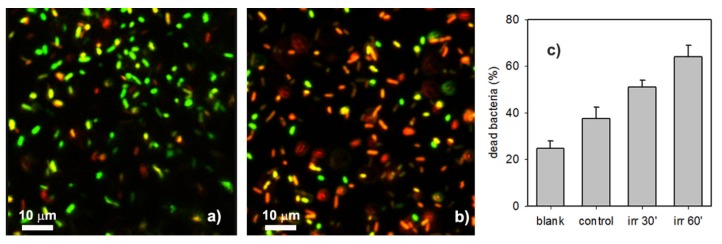
(**a**) Representative confocal image of bacteria inoculated on CuS NP monolayer on sample without irradiation; (**b**) the same on sample after 60’ of NIR irradiation (0.35 W/cm^2^); (**c**) percentage of dead bacteria found after a 3 h contact on a non-functionalized glass sample (blank), on a CuS NP-glass sample in absence of irradiation (control), and on a CuS NP-glass sample in presence of 30’ or 60’ irradiation within the 3 h contact. Field of view: 64.2 × 64.2 μm^2^.

Figure 5 represents typical results of the NIR induced antibacterial effect of CuS NP SAM. For the “control” CuS NP-glass non-irradiated sample (Figure 5a), a sensible number of dead bacterial cells (the red ones) can be observed (A_dead_/A_live_ = 0.6 ± 0.12, corresponding to 37.5% of dead bacteria) higher than the intrinsic mortality of bacteria seeded on non-functionalized blank substrates (A_dead_/A_live_ = 0.33 ± 0.06 on sterilized glass slides, corresponding to 24.8% of dead bacteria, see Figure S5 in Supporting Information). This is not surprising, as the whole experiment time (i.e., the whole contact time between bacteria and surfaces) takes about 3 h, and, as observed in the previous section, a microbicidal effect connected to sustained copper release is expected on such a timescale even in the absence of irradiation. We found that the NIR induced photo-thermal effect was efficient to kill bacteria on the glass NIR surface. NIR irradiation exposure for 30 min increased almost twofold the mortality of the bacteria (A_dead_/A_live_ = 1.05 ± 0.13 corresponding to 51% of dead bacteria). The bacteria mortality increases with irradiation time, reaching a value A_dead_/A_live_ = 1.77 ± 0.3 after 60 min, corresponding to 64% of dead bacteria (Figure 5b). The overall result is summarized in Figure 5c.

## 4. Conclusions

We demonstrated for the first time how some of the particular features of CuS NP can be exploited when these inorganic nano-objects are grafted onto a macroscopic surface. We obtained stable samples, showing no release of nano-objects in the environment and bearing a homogeneous monolayer of almost monodisperse spherical CuS NP. The preparation is easy and cost effective, and produces surfaces carrying an extremely low quantity of a material which can be considered safe and not harmful to the human body [22,27]. In the present study we did not perform toxicity tests, but previous studies indicated a low toxicity of CuS patterns on Human Dermal Fibroblasts cells [27], good biocompatibility for in vivo application [18], and good hemo-co-compatibility on human red blood cells [23]. Thus, CuS NP and limited Cu^2+^ release are considered safe for the human body, and this is strengthened by the discovery that laser-activable CuS NP are able to eradicate bacteria but also to release Cu^2+^ to accelerate wound-healing processes [20].

We want to stress again the fact that this is the first report in which a bulk surface has been rendered antibacterial simply by grafting of CuS NP, a safe, cost effective and easy to prepare nanomaterial. These surfaces possess a static antibacterial ability towards Gram- and Gram+ bacteria, which seems to be related to copper release in solution, especially active in the first 24 h, so covering the interval in which bacterial adhesion and reproduction are extremely critical for the spread of an infection on a medical surface. In addition, these samples can exert another microbicidal effect as a result of a photo-thermal effect that can be switched on demand by proper laser excitation, and our results suggest it could be used on surfaces of medical devices in those cases of particularly aggressive bacterial contaminations, to sustain the static antibacterial effect.

As in several systems prepared via a layer by layer approach [37,38,39,40], the proposed surface modification proceeds with a simple electrostatic deposition, in this case on an APTES functionalized glass surface. The approach proposed here may offer several advantages: CuS NP are easy to prepare and almost inexpensive, especially when compared to noble metal nanoparticles or synthetic antibacterial moieties that have to be grafted to surfaces. Coating realization is almost straightforward, does not involve difficult steps, and can be applied on every kind of surface. Glass-like SiO_2_ films can in fact be easily cast on a large spectrum of materials by gelification of a siloxane sol. Thus, the method here described, involving a first easy step of silanization of a glass sample, can be applied in principle to coat almost any kind of surface of medical use like wound dressings, needles, prosthetic and subcutaneous devices, surgical sutures, to name but a few examples. All these kinds of surfaces surely need antibacterial protection in the first hours of implant, here ensured by the ability of CuS NP to release small and controlled amounts of copper at the most critical phase of bacterial adhesion and proliferation, and additionally will profit by a switchable laser treatment which gives an “extra” elimination of bacteria cells, when needed.

## Figures and Tables

**Table 1:** Characterization parameter of CuS NP prepared under different citrate concentrations.

Citrate Concentration (M)	Z Potential (mV) ^1^	Diameter from DLS (nm) ^1^	LSPR Max (nm) ^1^
3.4 × 10^−3^	−22 (4)	5 (3)	936 (10)
7.0 × 10^−4^	−23 (5)	13 (2)	960 (10)
3.4 × 10^−4^	−25 (4)	20 (3)	980 (10)

^1^ Values represents the mean value of at least three preparations.

**Table 2:** Microbicidal effect (ME) values ^1^ for CuS NP-glass samples.

Contact Time	S. aureus	E. coli
5 h	1.7 (0.5)	1.9 (0.7)
24 h	3.2 (0.5)	3.2 (0.5)

^1^ Values represents the mean value of at least three experiments.

## Supplementary Materials

The following are available online at https://www.mdpi.com/2079-4991/10/2/352/s1, Figure S1: Raman spectra of glass slide, APTES-glass slide, colloidal CuS NP and of CuS NP–glass samples; Figure S2: SEM image of a glass slide functionalized with APTES. Figure S3: UV–Vis spectrum of a CuS NP-glass sample after one week of immersion in water, spectrum of a glass slide functionalized with APTES; Figure S4: thermogram obtained using irradiation at 950 nm with power set to 450 mW; Figure S5: confocal microscopy image of an experiment on *P. aeruginosa* cells inoculated on a blank glass and without NIR laser irradiation.
