# Supplementary material for: Self-Assembled Monolayers of Copper Sulfide Nanoparticles on Glass as Antibacterial Coatings
Source: Nanomaterials (Basel). 2020 Feb 18;10(2):352. doi: 10.3390/nano10020352 (PMC7075189; doi:10.3390/nano10020352)
Supplement: Supplementary file 1 [file nanomaterials-10-00352-s001.pdf]

Supplementary materials

Self-Assembled Monolayers of Copper Sulfide Nanoparticles on Glass as Antibacterial Coatings

Chiara Gargioni[1], Mykola Borzenkov[2], Laura D'Alfonso[2], Paola Sperandeo[3], Alessandra Polissi[3], Lucia Cucca[1], Giacomo Dacarro[1], Pietro Grisoli[4], PiersandroPallavicini[1], Agnese D'Agostino[1], Angelo Taglietti[1]*

[1] Department of Chemistry, University of Pavia, Viale Taramelli 12, 27100 Pavia , Italy; E-mail: angelo.taglietti@unipv.it
[2] Nanomedicine Center, Department of Physics, University of Milano-Bicocca, piazza dell'Ateneo Nuovo,20126 Milan, Italy
[3] Department of Pharmacological and Biomolecular Sciences, University of Milan, via Balzaretti 9, 20133 Milan, Italy
[4] Department of Drug Sciences, University of Pavia, Viale Taramelli 14, 27100 Pavia , Italy.
* Correspondence: angelo.taglietti@unipv.it; Tel.: +39-382-987342

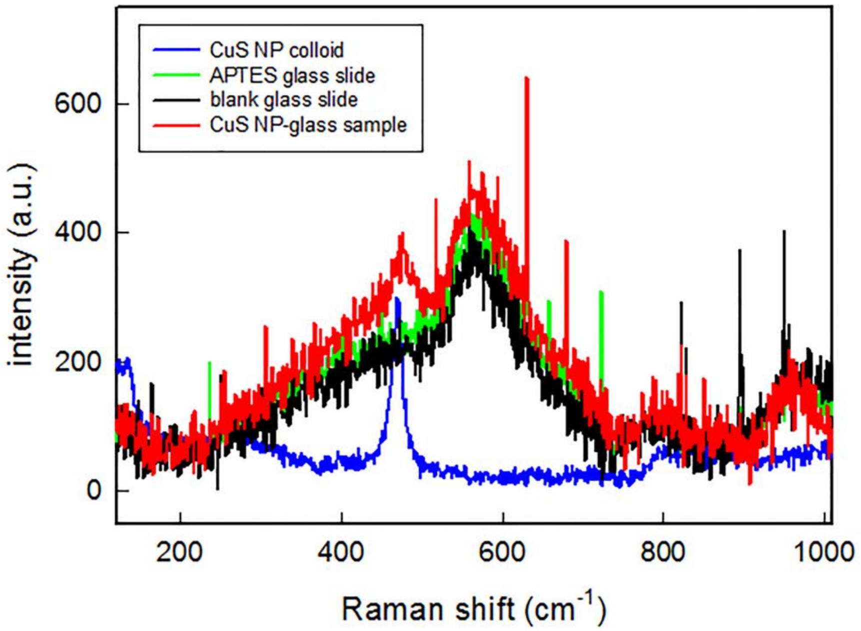

Figure S1 Raman spectra of a colloidal sample of CuS NP dried on glass (blue line), of a CuS NP-glass sample (red line), of a blank glass slide (black line), of an APTES functionalized glass slide (green line).

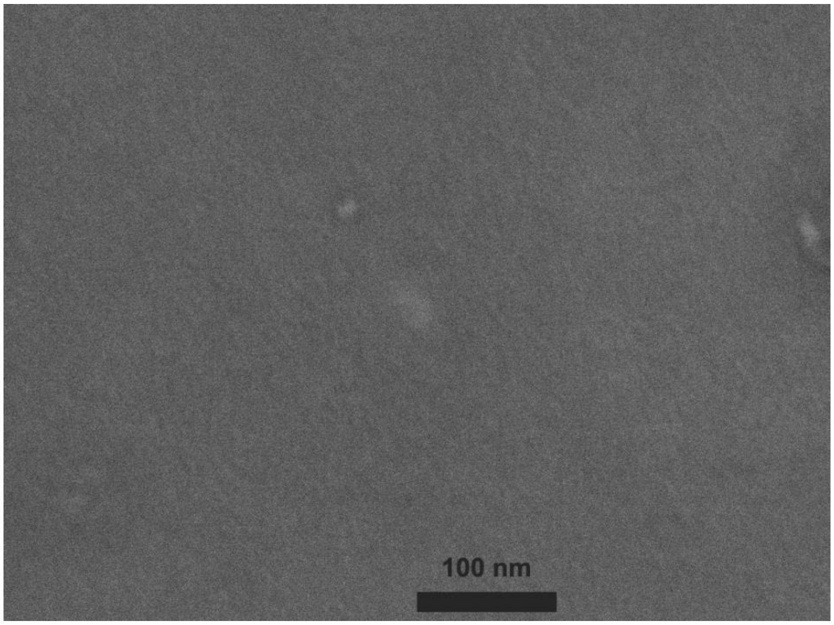

Figure S2 SEM image of a glass slide functionalized with APTES.

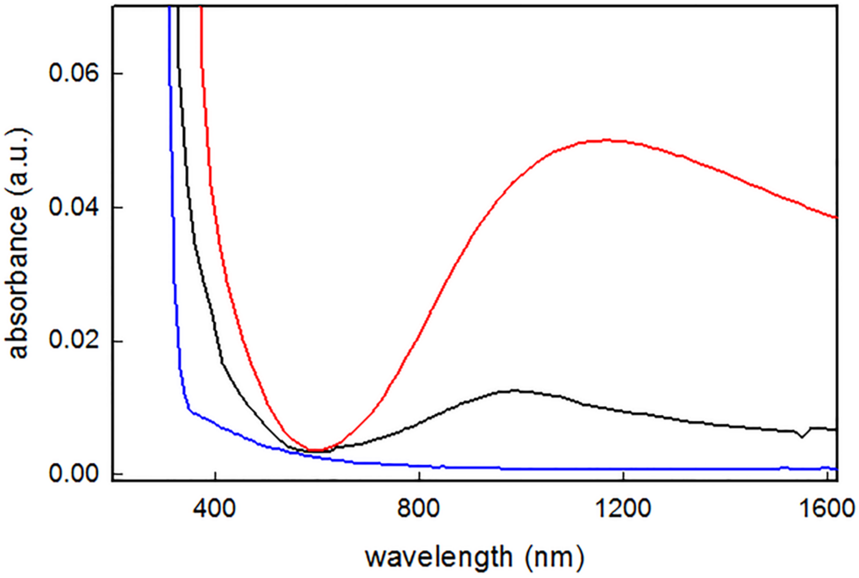

Figure S3 UV–vis spectrum of a CuS NP-glass sample freshly prepared (red line) compared with the spectrum of a CuS NP-glass sample after one week of immersion in water (black line). The blue line represents the spectrum of a glass slide functionalized with APTES.

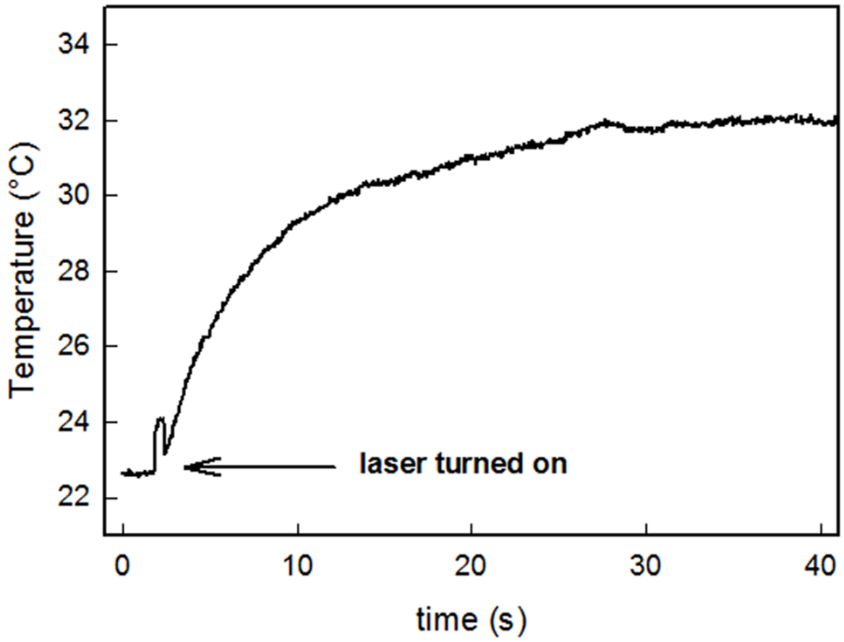

Figure S4 Temperature increase as a function of irradiation time, measured under NIR laser irradiation at 950 nm, 0.35 W/cm^2 of CuNP-glass sample

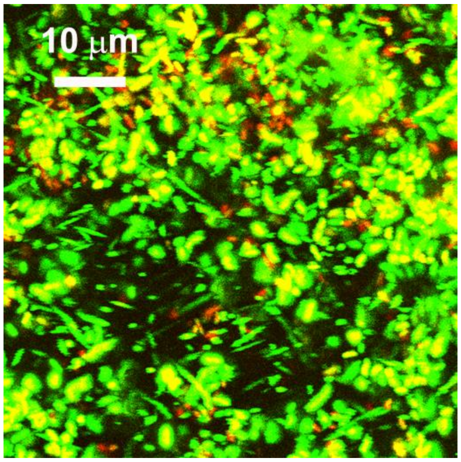

Figure S5 Representative confocal image of bacteria (*P. aeruginosa*) inoculated on blank sample without irradiation. The overall contact time between bacteria inoculation and image registration was of three hours. Field of view: 64.2x64.2 μm^2
